# UGT1A1 mutation association with increased bilirubin levels and severity of unconjugated hyperbilirubinemia in ABO incompatible newborns of China

**DOI:** 10.1186/s12887-021-02726-9

**Published:** 2021-06-01

**Authors:** Hui Yang, Fen Lin, Zi-kai Chen, Lin Zhang, Jia-Xin Xu, Yong-Hao Wu, Jing-Ying Gu, Yu-Bin Ma, Jian-Dong Li, Li-Ye Yang

**Affiliations:** 1 Department of Laboratory Medicine, School of Medicine, Yangtze University, Jingzhou, Hubei Province 434023 People’s Republic of China; 2 Central Laboratory, Chaozhou Central Hospital Affiliated to Southern Medical University, Chaozhou, Guangdong Province People’s Republic of China; 3 School of Food Engineering and Biotechnology, Hanshan Normal University, Chaozhou, Guangdong Province People’s Republic of China; 4 Department of Pediatrics, Chaozhou Central Hospital Affiliated to Southern Medical University, Chaozhou, Guangdong Province People’s Republic of China; 5 Lab for Respiratory Disease, People’s Hospital of Yangjiang, No. 42 Dongshan Road, Yangjiang, 529500 Guangdong Province People’s Republic of China

## Abstract

**Background:**

Neonatal hyperbilirubinemia causing jaundice is common in East Asian population. Uridine diphosphate glucuronosyltransferase isoenzyme (UGT1A1) glucuronidates bilirubin and converts the toxic form of bilirubin to its nontoxic form.

**Method:**

A retrospective study was conducted to review clinical information of ABO hemolysis neonates (ABO HDN) admitted to the Department of Neonatology, referred for neonatal hyperbilirubinemia, in a large general hospital of southern China from 2011 to 2017. Variation status of UGT1A1 was determined by direct sequencing or genotype assays.

**Result:**

Sixty-nine ABO HDNs were included into the final analysis. UGT1A1 c.211 G > A mutation (UGT1A1*6, p.Arg71Gly, rs4148323) was significantly associated with the increased bilirubin level in ABO HDNs, after adjusted by age, sex and feeding method (*P* = 0.019 for TBIL, *P* = 0.02 for IBIL). Moreover, heterozygous and/or homozygous UGT1A1 mutations in the coding sequence region were significantly associated with the increased risk of developing hazardous hyperbilirubinemia (as defined by TSB > 427 umol/L) as compared those with a normal UGT1A1 genotype (OR_adj_ = 9.16, 95%CI 1.99–42.08, *P* = 0.002) in the study cohort.

**Conclusion:**

UGT1A1 variant in coding region is actively involved in the pathogenesis of ABO hemolysis related neonatal hyperbilirubinemia. Genetic assessment of UGT1A1 may be useful for clinical diagnosis of neonatal unconjugated hyperbilirubinemia.

## Background

Neonatal hyperbilirubinemia causing jaundice is a complex pediatric disorder affecting up to 80% of newborns worldwide [1, 2]. Although it is benign in the vast majority of infants, total serum bilirubin (TSB) may accumulate and reach very high levels in some cases. Once it reaches the hazardous threshold levels, certain brain regions can be irreversibly damaged [3–5].

In 2004, the American Academy of Pediatrics (AAP) guidelines listed the East Asia, including mainland China as a major risk factor for severe hyperbilirubinemia [6]. The incidence and severity of neonatal hyperbilirubinemia in Asians and American Indians are much higher, as compared to those in Caucasian and black populations. It has been suggested that the high incidence rate of hemolytic anemia, caused by ABO alloimmunization or glucose-6-phosphate dehydrogenase (G6PD) deficiency, may predispose these populations to neonatal hyperbilirubinemia. The overall risk dramatically increased for a TSB level of 20 mg/dL (342 mmol/L) [7].

Congenital variation of the bilirubin clearance rate in the liver is also the biological basis of neonatal hyperbilirubinemia risk in Asia. The key bilirubin metabolism gene, namely, the hepatic bilirubin conjugating isoenzyme UDP glucuronosyltransferase family 1 member A1 (UGT1A1) was classically described for Crigler-Najjar type I and II syndrome as well as Gilbert syndrome [8–10]. More and more evidence has shown that the genetic variation of UGT1A1 is also closely related to the incidence rate and severity of neonatal hyperbilirubinemia [10–14]. However, the innate variants of the UGT1A1 gene are under-diagnosed in neonates and under-recognized as a cause of severe hyperbilirubinemia clinically.

In our previous studies [14, 15], we have demonstrated the role of UGT1A1 in non-hemolytic unconjugated hyperbilirubinemia in Chinese newborns. Here, we aim to further explore the role of UGT1A1 variants in ABO hemolytic disease of newborns (ABO HDNs). We suspected that ABO HDNs that carried the gene variant for Gilbert’s syndrome may have a higher risk of developing severe hyperbilirubinemia. This study may enhance our understanding of the genetic basis of neonatal hyperbilirubinemia in Asia.

## Methods

### Participants and sample collection

This retrospective study was conducted in the pediatric center of Chaozhou Central Hospital affiliated to Southern Medical University, Chaozhou, China. All neonates enrolled in this study were admitted to the study center from 2011 to 2017. Demographic and clinical records and laboratory results were reviewed and collected by the ordering physicians from electronic medical records.

Information recorded included date of birth, gender, weight at birth, mode of delivery, gestational age, feeding mode, Apar score symptoms, signs, laboratory findings, medical records and underlying comorbidities. Laboratory tests including the TSB level, G6PD enzyme assay, and three serological tests for neonate hemolytic disease were specially recorded. Neonates without complete medical information were excluded from final analyzed.

The study group comprised infants of blood group A or B with a blood-group-O mother (ABO incompatible) suffering from neonatal hemolytic disease confirmed by seroimmunity antibody tests. Newborns with birth weight less than 2500 g and gestational age less than 37 weeks were excluded. In addition, the infants who had other risk factors for jaundice were also excluded: maternal diabetes, infection, Rh incompatibility-caused hemolytic disease, asphyxia, G6PD deficiency, hypothermia, drug treatment, cerebral hematoma, dehydration, metabolic diseases, hypothyroidism, liver disease, and major organ abnormalities. These conditions were determined by past and family history, as well as clinical and laboratory tests. The information was reviewed and retrieved from the electronic medical records.

All the laboratory tests were conducted by our clinical laboratory staff according to National Clinical Laboratory Procedures. The serum bilirubin level was measured using a commercial TBIL/IBIL assay kit (KEFANG biotech, China, Co, Ltd) by automatic biochemical analytic method. Three serological antibody tests were done using commercial three-cell panel (LIBO biotechology, China, Co, Ltd) by gel technique. Three tests including red blood cells direct antiglobulin test (direct Coombs test), free antibody test (free) and antibody release test were performed according to the manufacturer’s protocol.

Hyperbilirubinemia was diagnosed according to the updated clinical guidelines of the Chinese Medical Association for neonates [16]. Serological diagnostic criteria for ABO hemolysis were as follows: 1) confirmed cases were neonates with two positive results of the three tests or the result of antibody elution test proved to be positive; 2) suspected cases were neonates only positive for either direct Coombs test or serum free antibody test. Antibody elution test was the final confirmed diagnosis for neonatal hemolysis disease.

After clinical diagnosis, the EDTA anticoagulant whole blood samples were collected prospectively and stored at − 20 °C prior to UGT1A1 genotyping.

This study was initially approved by the Ethics Committee of Chaozhou Central Hospital in 2011 (No. 2011021), and then the second ethical approval was obtained in 2015 (No.2015001). As the patients data were analyzed anonymously, and the blood samples in this study were used after the clinical diagnosis (blood routine examination), a waiver of written consent was approved by the Ethics Committee of Chaozhou Central Hospital. Our group had the administrative permissions to access the data according to associated regulation by national health commission of P.R.China. Specially, clinical data were collected by pediatrician, laboratory data was reviewed by the clinical laboratory staff.

### DNA extraction and UGT1A1 genotyping

Genomic DNA from peripheral blood specimens was extracted with FlexiGene DNA Kit (Qiagen Inc., Valencia, California). The DNA sequences of promoter, exons, and exon-intron boundaries of UGT1A1 were determined by polymerase chain reaction (PCR) and DNA sequencing as previously described [14]. The repetitive polymorphism of (TA)_n_ in the promoter region was further confirmed by capillary electrophoresis, described in detail in our previous studies [14, 15].

### Data analysis

Hardy-Weinberg equilibrium (HWE) was used to test the two common variants of UGT1A1 locus. Linkage disequilibrium (LD) analysis for the polymorphisms within UGT1A1 was performed, and the haplotypes were inferred using the web tool SNPStats (http://bioinfo.iconcologia.net/SNPStats), [17] as described in our previous studies [14, 15].

The differences of categorical variables between the two groups were compared by chi-square test or Fisher exact test. According to the UGT1A1 genotype, all infants were divided into two (wild type and mutant) or three groups (wild type, heterozygous mutant, and homozygous mutant). Independent group t-test was used to analyze the difference of continuous variables if the dataset was normally distributed; otherwise, the Mann-Whitney test was used.

After adjusting for known clinical risk factors for neonatal hyperbilirubinemia (including gender, breastfeeding, and age), a linear regression model was used to assess the association between specific polymorphisms or haplotypes of UGT1A1 and the TSB peak values prior to phototherapy. According to the AAP guidelines, all the infants were divided into the hazardous group (TSB ≥ 427 μmol/L), the severe group (TSB ≥ 342 μmol/L), and the non-severe group (TSB < 342 μmol/L). Then logistic regression was used to evaluate the association of the UGT1A1 gene variations with the severity of hyperbilirubinemia.

All statistical analyses were performed using the two-sided test by SPSS (version 16) and SNPstat, and *P* < 0.05 was considered as statistically significant.

## Results

### Clinical analysis

After excluding neonates with the conditions described above, a total of 69 full-term ABO HDNs infants were admitted to the hospital on the 3rd day (median) after birth (range, 1–10 days). They are all Han ethnicity from southern China. All the studied neonates presented with skin jaundice and higher TcB level. The average peak serum total serum bilirubin level (TBIL) was 335 μmol/L (123–652 μmol/L). Among 69 ABO HDNs, 31 cases had peak TSB ≥ 342 μmol/L, in which 15 neonates had TSB ≥ 427 μmol/L. All the neonates received phototherapy. Most of the neonates were discharged without complication, except for two infants with bilirubin encephalopathy symptoms, showing high-pitched cry, lethargy, and poor sucking, and loss of upward gaze. The MRI showed high T2 signal in the globus pallidus.

There were significant differences in the bilirubin levels, but no differences in average gestational age, birth weight, gender and feeding pattern between the two groups of neonates divided according to the c.211 genotypes of UGT1A1-- the most common UGT1A1 variant in Asian population (Table 1).

**Table 1:** Demographic and clinical features among the neonates with ABO hemolytic disease (ABO HDN) in UGT1A1 c. 211 G > A mutation group VS c.211 normal group (*N* = 69)

	c.211 mutation/ABO HDN	c.211 normal /ABO HDN	***P***
**Sex**			NS
**Male**	6(30.0)	25(51.0)	
**Female**	14(70.0)	24(49.0)	
**Gestational week**	39.4 ± 0.2	39.2 ± 0.1	NS
**Birth weight (kg)**	3.3 ± 0.08	3.2 ± 0.06	NS
**Peak serum bilirubin levels (umol/L)**			
**TBIL**	404(219–556)	315(146–585)	0.028
**IBIL**	380(201–528)	303(129–552)	0.037
**DBIL**	14 (5.0–69)	12(3–35)	NS
**Feeding**			NS
**Breast fed**	3(15.0)	11(22.4)	
**Breast and formula**	2(10.0)	9(18.4)	
**Formula**	14(70.0)	20(40.8)	
**Unknown**	1(5.0)	9(18.4)	
**Delivery method**			NS
**Vaginal**	7(35.0)	27(55.1)	
**Cesarean**	13(65.0)	22(44.9)	

Data are presented as n (%), mean standard deviation, or median (95% Confidence Interval)

NS: no significance

### UGT1A1 variant results

In addition to the two common variants of UGT1A1 gene, TA7 polymorphism (UGT1A1*28, rs8175347) in the promoter and c.211 G > A mutation (UGT1A1*6, p.Arg71Gly, rs4148323) in exon 1, another coding variant c.1091C > T (UGT1A1*73, p.Pro364Leu, rs34946978) was observed in the neonates. Specifically, heterozygote of TA7 promoter polymorphism (TA6/TA7) was detected in 9 neonates, with no homozygote for TA7 polymorphism (TA7/TA7) observed. The frequency of heterozygous (G/A) and homozygous (A/A) genotypes of c.211 G > A mutation were 0.275 (19/69) and 0.014 (1/69), respectively. Three cases were observed with heterozygous c.1091C > T mutation (Table 2).

**Table 2:** Minor allelic, genotypic, and haplotype distributions of UGT1A1 polymorphism in studied patients (*N* = 69)

UGT1A1 polymorphism	Location	N	Frequency	***P***_**H-W**_^**a**^
**TATA box (rs8175347)**	promoter			1
**TA7**		9	0.07	
**TA6**		129	0.93	
**TA7/TA7**		0	0	
**TA6/TA7**		9	0.13	
**TA6/TA6**		60	0.87	
**C.211 G > A (rs4148323)**	Exon 1			1
**A**		21	0.15	
**G**		117	0.85	
**A/A**		1	0.01	
**G/A**		19	0.28	
**G/G**		49	0.71	
**c.1091 C > T (rs34946978)**	Exon 4			1
**T**		3	0.02	
**C**		135	0.98	
**T/T**		0	0	
**C/T**		3	0.04	
**C/C**		66	0.96	
**Haplotype (rs8175347-rs4148323-rs34946978)**				
**TA6GC**			0.76	
**TA6AC**			0.15	
**TA7GC**			0.065	
**TA6GT**			0.018	
**TA6AT**			0.0037	

^a^Hardy-Weinberg Equilibrium test *p* value

There was a strong pairwise LD between the UGT1A1 promoter polymorphism and exon mutation (|D|’ > 0.8), but none of the polymorphisms in our study had a statistically significant deviation in the HWE test. Haplotype analysis showed that the TA6GC (rs8175347-rs4148323-rs34946978) was the predominant haplotype among the study subjects (75.9%), (Table 2).

### Co-inherited UGT1A1 variant on bilirubin levels in ABO HDNs

**Fig. 1:**
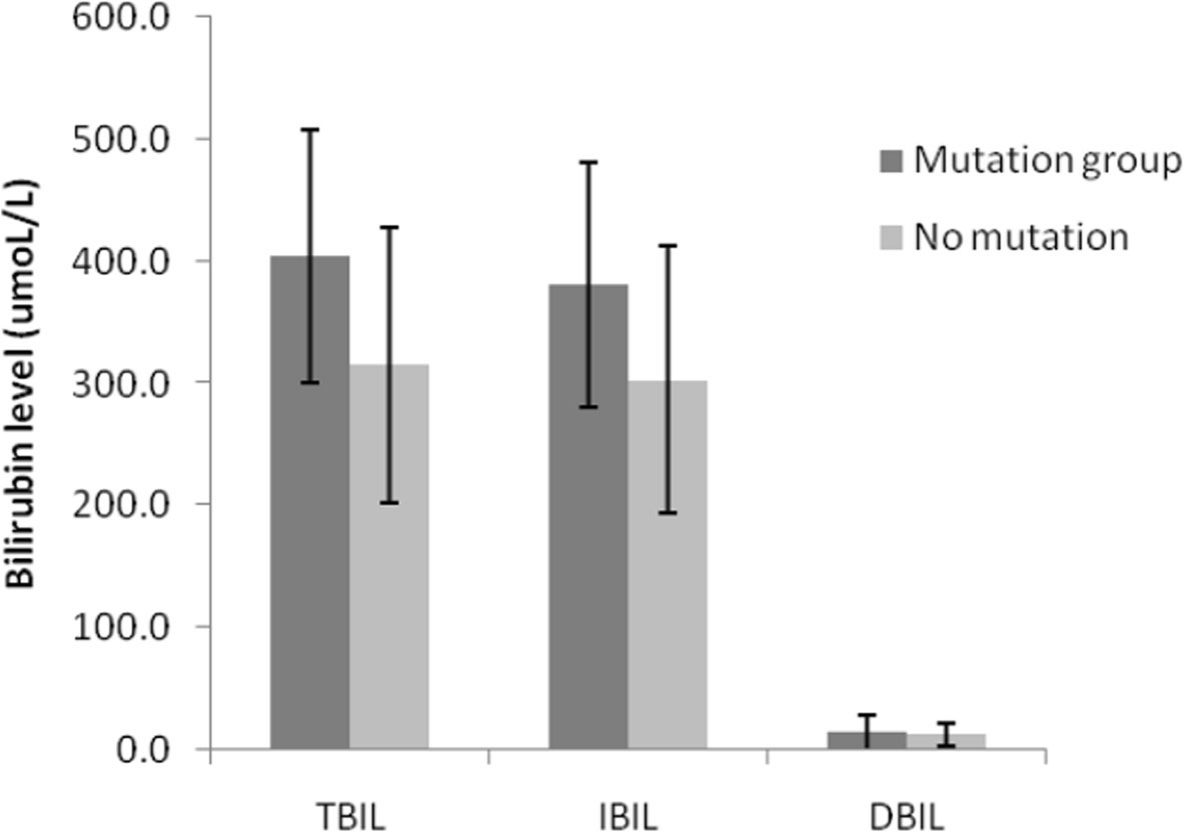
Distribution of serum bilirubin levels among the subgroup of the ABO hemolytic disease neonates according to UGT1A1 c.211 genotypes

When analyzing the peak bilirubin levels according to UGT1A1 genotypes, the average peak bilirubin levels (TBIL and indirect serum bilirubin level (IBIL)) of ABO (+) neonates with both heterozygous and homozygous c.211 G > A coding mutation were higher as compared to those with normal UGT1A1 genotype (*P* = 0.03 for TBIL, *P* = 0.04 for IBIL), whereas direct serum bilirubin level (DBIL) showed no statistical difference among the three genotypes (Table 1, Fig. 1). No significant difference in bilirubin levels was observed in the presence of either heterozygous of TA7 promoter polymorphism or heterozygous of c.1091C > T mutation in the neonates.

After adjusting the potential covariance (age, gender, and feeding method), c.211 G > A mutation was still associated with the increased bilirubin levels (OR_adj_ = 78.2, 95%CI 14.7–141.8, *P* = 0.019 for TBIL, OR_adj_ = 73.3, 95%CI 12.8–133.7, *P* = 0.021 for IBIL) (Table 3). Moreover, haplotype association analysis showed that the TA6AC (rs887829-rs4148323- rs34946978) was significantly associated with increased bilirubin levels (OR_adj_ = 84.0, 95%CI 23.2–144.8, *P* = 0.009 for TBIL, OR_adj_ = 79.0, 95%CI 21.4–136.6, *P* = 0.009 for IBIL). Haplotype TA6GT also showed significant association with increased bilirubin levels (OR_adj_ = 107.9, 95%CI 93.7–122.1, *P* < 0.001 for TBIL, OR_adj_ = 107.0, 95%CI 93.6–120.4, *P* < 0.001 for IBIL) (Table 3).

**Table 3:** The associations between serum bilirubin level and different types of UGT1A1 mutation and genotypes adjusted by age, gender, and feeding practice: Linear regression analysis (*N* = 59^a^)

Genotypes	TBIL		IBIL	
	OR_**adj**_^b^ (95%CI)	***P***	OR_**adj**_^b^ (95%CI)	***P***
**TATA box**				
**TA6/TA6**	0.00	0.57	0.00	0.58
**TA6/TA7**	30.0(−73.1–133.2)		27.6(−70.4–125.5)	
**c.211 G > A**				
**G/G**	0.00	0.019	0.00	0.02
**G/A-A/A**	78.2(14.7–141.8)		73.3(12.8–133.7)	
**c.1091 C > T**				
**C/C**	0.00	0.16	0.00	0.14
**C/T**	103.1(−39.8–245.9)		103.6(−31.7–238.9)	
**Haplotype**^**c**^ **(Frequency)**				
**TA6GC(75.85%)**	0.00		0.00	
**TA6AC(16.53%)**	84.0(23.2–144.8)	0.0092	79.0(21.4–136.6)	0.0097
**TA7GC(5.08%)**	70.4(−26.8–167.7)	0.16	65.6(−26.6–157.9)	0.17
**TA6GT(2.12%)**	107.9(93.7–122.1)	< 0.0001	107.01(93.6–120.4)	< 0.0001
**TA6AT(0.42%)**	149.9(147.3–152.6)	< 0.0001	152.4(150.0–154.8)	< 0.0001

^a^ Ten cases with the feeding practice unknown were not taken into the regression analysis

^b^Adjusted for age, gender, and feeding practice

^c^polymorphisms are in order of: rs8175347-rs4148323-rs34946978

### Co-inherited UGT1A1 variant on severe hyperbilirubinemia risk in ABO HDNs

The incidence rates of hazardous and severe hyperbilirubinemia in the ABO HDNs were compared in different types of UGT1A1 genotype. Promoter polymorphism and exon mutations were analyzed, separately. Compound heterozygous mutations in the coding sequence were regarded as homozygous mutations. No statistical difference of severe hyperbilirubinemia incidence was found between ABO HDNs with and without the UGT1A1 mutation (*P* > 0.05). On the contrary, after adjusted by age, gender, and feeding method, ABO HDNs with heterozygous and/or homozygous mutations in the UGT1A1 coding sequence region had a relatively higher risk of developing hazardous hyperbilirubinemia than those with a normal UGT1A1 genotype (OR_adj_ = 9.16, 95%CI 1.99–42.08, *P* = 0.002). Moreover, haplotype association analysis showed that TA6AC was significantly associated with a higher incidence of hazardous hyperbilirubinemia in ABO HDNs (OR_adj_ = 9.41, 95%CI 1.80–49.26, *P* = 0.011) (Table 4).

**Table 4:** The associations between risk of severe neonatal hyperbilirubinemia and UGT1A1 coding sequence variants and different type of UGT1A1 haplotype in neonates with ABO hemolysis disease: multivariate logistic regression analysis (*N* = 59^a^)

Genotype	Total	TBIL > 342 umol/L			TBIL > 427umol/L		
	N(%)	N(%)	OR_**adj**_^b^ (95%CI)	***P***	N(%)	OR_**adj**_^b^ (95%CI)	***P***
**Model 1(*n*** **= 59)**							
**Wild type**	38(64.4%)	16(57.1%)	1.00		4(30.8%)	1.00	
**Heterozygous mutation**	19(32.2%)	10(35.7%)	2.25(0.66–7.61)	0.076	8(61.5%)	8.74(1.86–41.09)	0.008
**Homozygous mutation**	2(3.4%)	2(7.1%)	NA(0.00-NA)		1(7.7%)	15.09(0.61–375.49)	
**Model 2(*n*** **= 59)**							
**Wild type**	38(64.4%)	16(57.1%)	1.00		4(30.8%)	1.00	
**Heterozygous + Homozygous mutation**	21(35.6%)	12(42.9%)	2.72(0.82–9.03)	0.093	9(69.2%)	9.16(1.99–42.08)	0.002
**Halplotype (Frequency)**							
**TA6GC(75.89%)**			1.00			1.00	
**TA6AC(16.58%)**			3.46(0.97–12.39)	0.062		9.41(1.80–49.26)	0.011
**TA7GC(5.08%)**			2.57(0.37–17.89)	0.35		8.18(0.80–83.65)	0.083
**TA6GT(2.17%)**			2.38(0.12–48.13)	0.57		10.64(0.44–254.56)	0.15
**Other**^**c**^			–	–		–	–

^a^ Ten cases with the feeding practice unknown were not taken into the regression analysis

^b^Adjusted for age, gender, and feeding practice

^c^ Other haplotypes had frequencies less than 1%

## Discussion

Hyperbilirubinemia is a common disorder among infants. Infants in Asia, including China where hazardous hyperbilirubinemia is not rare [1, 18], are at a greater risk of developing hyperbilirubinemia. ABO incompatibility, one of the main causes of hemolytic disease in newborns [19], has been well documented to be associated with the incidence and severity of neonatal hyperbilirubinemia [7, 20]. It is estimated that 27% of newborns have ABO incompatibility in China, while only 15% worldwide [21]. Indeed, ABO hemolytic disease is regarded as an important factor in neonatal hyperbilirubinemia in East Asia [7].

The serum bilirubin level is a consequence of many factors, which may change the production and excretion of bilirubin. Currently, more and more attention has been paid to the contribution of genetic polymorphisms of the bilirubin clearance genes in the pathogenesis of hyperbilirubinemia. In this study, we demonstrated that UGT1A1 mutation and polymorphism play an active role in the pathogenesis of ABO hemolysis-related neonatal hyperbilirubinemia.

The UGT1A1 coding sequence variant c.211 G > A (UGT1A1*6, G71R), the main cause of Gilbert syndrome in East Asia, was also the predominant association factor with high TSB levels and neonatal hyperbilirubinemia risk in the Asian population without any additional icterogenic factors [14, 15, 22–25]. In this study, we further confirmed that both the occurrence rate and bilirubin levels of hyperbilirubinemia in the ABO HDNs were significantly higher in the presence of homozygous or heterozygous c.211 mutation. One recent study in Chinese neonates also reported the contribution of c.211 mutation to neonatal hyperbilirubinemia risk in ABO HDN patients [26]. However, a similar study in Turkish neonates failed to discern the association of c.211 variant with the increased hyperbilirubinemia risk in ABO HDNs [27]. The discrepancy may be due to the fact that the research subjects are from different races and different regions. Large-scale studies across different ethnic groups and regions are necessary to draw further conclusions.

The polymorphism of (TA)_n_ repeat in the UGT1A1 promoter region has also been widely studied. TA7 is common in European and African populations, and it was proposed to be the genetic basis for Gilbert syndrome of Caucasians [28]. However, increasing studies in China and other countries reported that TA7 promoter polymorphism was not directly related to neonatal hyperbilirubinemia in most Asian regions [22]. More interestingly, several recent studies in Asian populations, including our previous studies, have shown that heterozygous of TA7 promoter may not cause neonatal hyperbilirubinemia, and may even have a protective effect [14, 15, 29–31]. In this study, we also observed that co-expression of TA6 allele, but not TA7, with the exon mutation (rs8175347-rs4148323-rs34946978: TA6AC/TA6GT) in UGT1A1 gene was associated with increased bilirubin levels and neonatal hyperbilirubinemia risk. This finding is contrary to previous studies in Caucasian populations and also inconsistent with the findings in Turkish neonates [27]. The reason for this contradictory effect of the UGT1A1promoter polymorphism in serum bilirubin level and neonatal hyperbilirubinemia risk is yet unknown.

Another coding sequence variants, UGT1A1*73(c.1091C > T, p.Pro364Leu, rs34946978), has also been reported to be linked to a significant decrease in UGT1A1 enzyme activity and the severity of Gilbert’s syndrome in both Caucasian and Asia populations [32, 33]. Although only the heterozygous c.1091C > T variant was identified in the present study, it has shown to increase bilirubin levels and hyperbilirubinemia risk in ABO HDNs in combination with other variant alleles of UGT1A1 genes (Table 3, Table 4).

There were several limitations in this study. Firstly, the size of the cohort was small, which may be the reason that some analyses could not reach statistical significance. Secondly, the newborns were all from one hospital in China. A larger multi-center study is necessary for future studies. Thirdly, it may not be comprehensive to analyze UGT1A1 alone. Evaluation of additional genes may also help to assess the genetic causes of unconjugated hyperbilirubinemia in newborns.

## Conclusion

Our study demonstrated that UGT1A1 variants contributed to the increased bilirubin level and risk of developing hazardous neonatal hyperbilirubinemia in ABO HDNs. It is actively involved in the pathogenesis of ABO hemolysis-related unconjugated hyperbilirubinemia. This association may caution clinicians to assess UGT1A1 variations for neonates with ABO hemolysis, and may aid in the identification of high-risk population, which is important for management and intervention of hazardous hyperbilirubinemia.

## Data Availability

The datasets used and/or analysed during the current study are available from the corresponding author on reasonable request.

## Abbreviations

ABO HDNs: ABO hemolytic disease of newborns
UGT1A1: Uridine diphosphoglucuronosyl transferase 1A1
TSB: Total serum bilirubin level
TBIL: Total serum bilirubin level
DBIL: Direct serum bilirubin level
IBIL: Indirect serum bilirubin level
